# Educational intervention in Primary Care for the prevention of congenital syphilis^1^

**DOI:** 10.1590/1518-8345.1612.2845

**Published:** 2017-01-30

**Authors:** Flaviane Mello Lazarini, Dulce Aparecida Barbosa

**Affiliations:** 2 PhD, Assistant Professor, Departamento de Saúde Coletiva, Universidade Estadual de Londrina, Londrina, PR, Brazil.; 3 PhD, Associate Professor, Escola Paulista de Enfermagem, Universidade Federal de São Paulo, São Paulo, SP, Brazil.

**Keywords:** Syphilis, Congenital, Treponemal Infections, Education, Continuing, Epidemiological Surveillance

## Abstract

**Objectives:**

to evaluate the efficiency of educational interventions related to the knowledge of health care professionals of Primary Care and to verify the impact on the vertical transmission rates of congenital syphilis.

**Method:**

a quasi-experimental study conducted in the city of Londrina, Paraná, between 2013 and 2015. An educational intervention on diagnosis, treatment and notification was carried out with 102 professionals with knowledge measurement before and after the intervention. Incidence and mortality data from congenital syphilis were taken from the system for notifiable diseases (SINAN) and the Mortality Information System (SIM). Excel tabulation and statistical analysis was done in the Statistical Package for Social Sciences, version 2.1. A descriptive and inferential analysis was performed.

**Results:**

the mean number of correct responses increased from 53% to 74.3% after the intervention (p < 0.01). The adherence to professional training was 92.6%. There was a significant reduction in the vertical transmission rate of syphilis from 75% in 2013 to 40.2% in 2015. In 2014 and 2015 there were no records of infant mortality from this condition.

**Conclusion:**

the educational intervention significantly increased the knowledge of health professionals about syphilis and collaborated to reduce the rate of vertical transmission of the disease.

## Introduction

According to the World Health Organization (WHO), in 2008, 1.4 million pregnant women worldwide were infected with syphilis, of which 80% had attended prenatal care. In that same year, about one fifth (20%) of these pregnant women did not attend the referral service to receive adequate prenatal care^1^.

Syphilis is a notifiable disease, which when transmitted intrauterus causes congenital syphilis presents up to 40% mortality rate. In untreated pregnant women the transmission is 70 to 100%, in the primary and secondary stages of maternal disease^2^. 

In view of these points, regarding the reemergence of syphilis in the general population and its range encompassing maternal and child health, as well as the difficulties encountered by epidemiological surveillance in overcoming the biomedical model, the fragmentation of care^3^ and the use of health policies established at the global and national levels, there is a need for strategic regional studies to enable intervention measures that are more effective and based on local reality. 

The Brazilian National Health System (SUS) recommends preventing the occurrence of this sentinel event^4^ and provides free diagnosis and treatment for the population, with emphasis on public policies aimed at pregnant women and their sexual partners. However, there is an increasing number of cases of congenital syphilis^1^, fetal deaths, abortions and several irreversible sequelae for newborns^2^ from this preventable infection. The objective of this study was to evaluate the efficiency of the educational proposal in the knowledge of health professionals about syphilis.

## Material and Method

Ethical aspects: the study was preceded by the approval of the Research Ethics Committee (CEP) of Unifesp (nº 520.189) and the signing of the free and informed consent form by the professionals included in the research.

Design, place and period: a quasi-experimental study with a “before and after” design carried out in the municipality of Londrina, Paraná, from October 2013 to December 2015.

Population and sample: every health professional working in Primary Health Care (AB) or in maternal and child care services, in coordination positions, who agreed to replicate the workshop in the health service where they worked, were invited to participate in the study. In this way 102 professionals, who were qualified and became local facilitators, composed the sample.

Data collection instruments: the material used to evaluate educational intervention was a self-administered structured questionnaire that was designed to be answered before and immediately after the permanent education workshops. Divided into two parts, the first with socio-demographic variables and training of professionals, and the second part with specific questions about the professionals’ knowledge regarding the recommendations of the Brazilian Ministry of Health (MS) for the prevention of vertical transmission of syphilis, fitting treatment strategy and follow-up of newborns with congenital syphilis. A pilot test of the questionnaire was conducted with six Primary Care professionals as a way to improve the collection instrument.

Incidence rates were monitored in epidemiological data from the Notifiable Disease Information System (SINAN) databases. For the data on fetal and infant mortality, the databases of the Mortality Information System (SIM) were used. All data were provided by the epidemiology sector of the Municipality of Londrina.

### Detail of the intervention, study variables and data analysis

The theme of the adequate use of the protocols instituted by the MS (Rede Cegonha - Stork Network and Rede Mãe Paranaense - Paranaense Mom’s network) for the prevention, diagnosis and treatment of congenital and acquired gestational syphilis (in the case of sexual partnerships) was addressed in the permanent education activities. The intervention was organized in partnership with the working group of the Observatory for Syphilis comprising doctors (a gynecologist who attends in the Primary Care and acts in the regional health management and Pediatric and Infectious diseases specialist that operates in the epidemiological sector), as well as nurses who are in the Women’s Health, Municipal Maternity, Municipal Maternal and Child Mortality Prevention Committee (CMPMMI) and the Municipal Testing and Counseling Center (Brazilian health service that carries out diagnostic and prevention of sexually transmitted diseases).

In order to attend all 54 Health Units of the Municipality, the training of these professionals was divided into three stages, the first of which took place in December 2013, the second in June 2014 and the third in September 2014. Professionals trained as Facilitators implemented two flowcharts descriptors of the work process in health services. The First Flow details the process of diagnosis of syphilis during the prenatal (PN). The second flow is used when there is confirmation of syphilis. The workshop participants built a Work Process Matrix in each Basic Health Unit (UBS) - a primary care service in Brazil that aims to protect community health, prevent diseases, diagnose, treat, rehabilitate and reduce damage - with its health teams, reinforcing the importance of quality PN and the responsibility of all in preventing disease and sequelae for future babies.

The variables studied were divided into socioeconomic and professional characteristics of the participants of syphilis diagnosis and management workshops: age, sex, graduation (medicine, nursing, nursing technicians, other undergraduate), place of work (primary care, other services), period of training in years, duration of maternal-infant care work in years, duration of diagnosis and management of syphilis work in years; participants’ knowledge on syphilis: Difference between FTA-Abs and VDRL serology (treponemal and non-treponemal test); Number of mandatory VDRL exams in PN according to the Mãe Paranaense Network; Appropriate conduct when the VDRL test is reagent; Appropriate management when the diagnosis of gestational syphilis is confirmed; Procedures for the control and follow-up of gestational syphilis; Syphilis notification; Adequate therapeutic regimen according to the staging of syphilis (primary, secondary and tertiary); Monitoring of the newborns diagnosed with syphilis during pregnancy.

Analysis of results and statistics: a descriptive analysis of the variables was performed through the mean, absolute and relative frequencies. The evaluation of the questionnaire was carried out taking into account the correct and incorrect answers according to the reference that supported the training. The McNemar test for correlated frequencies was used as the inferential analysis. Data was tabulated in Excel for Windows^®^ spreadsheets, version 2010 and analyzed by the software *Statistical Package for Social Sciences* ^®^ 21. The values of descriptive levels equal to or lower than this value (p ≤ 0.05) were considered statistically significant.

## Results 

The results related to the characterization of health professionals who participated in the permanent education workshops, and became facilitators in the Basic Health Units (UBS), are presented in Table 1 below.

**Table 1:** Sociodemographic and professional characterization of the participants of the permanent education workshops for the diagnosis and management of syphilis, Londrina, PR, Brazil, 2014

Characteristics	Description	N*(102)						%
		Female	Male	Total		Female	Male	Total
Age group and sex (n = 90)	22-30	18	02	20		23.1	16.7	22.2
	31-40	28	06	34		35.9	50.0	37.8
	41-50	14	-	14		17.9	-	15.6
	51-61	18	04	22		23.2	33.3	24.4
Graduation	Medicine	14	07	21		16.3	43.8	20.6
	Nursing	64	08	72		74.4	50.0	70.6
	Nursing technician	07	01	08		8.1	6.3	8.1
	Other Graduation	01	-	01		1.2	-	0.7
Location	Primary Care	79	14	93		91.9	87.5	91.1
	Other Services	07	02	09		8.1	12.5	8.9
Training time (in years)	< 1	06	-	06		7.0	-	5.9
	1 a 3	11	02	13		12.8	12.5	13.7
	4 a 5	04	02	06		4.7	12.5	5.9
	6 a 10	17	04	21		19.8	25.0	19.6
	> 10	48	08	56		55.8	50.0	54.9
Time of action in maternal and child care (in years)	< 1	07	-	07		8.1	-	6.9
	1 a 3	20	03	23		23.3	18.8	22.5
	4 a 5	06	04	10		7.0	25.0	09.9
	6 a 10	14	01	15		16.3	6.3	14.7
	> 10	39	08	47		45.3	50.0	46.0
Time working in the diagnosis and management of syphilis (in years)	< 1	08	-	08		9.3	0.0	7.8
	1 a 3	33	04	37		38.4	25.0	36.3
	4 a 5	04	04	08		4.7	25.0	07.8
	6 a 10	15	03	18		17.4	18.8	17.7
> 10	26	05	31		30.2	31.3	30.4

* When the number of responses was lower than the number of people who answered the questionnaire the total was specified below the variable

Within a total of 102 study participants, majority female, with graduation in nursing and acting in Primary Care. Of the 54 UBS represented, 92.6% (50) undertook training with their health teams. The participants’ ages ranged from 22 to 61 years and the median age was 38 years. Most of the respondents stated that they had more than 10 years of practice, both in the time of training and in the time working in maternal and childcare. In spite of this, 36.3% answered that their work period in syphilis management was from one to three years.

Table 2 shows the distribution of participants’ answers about the diagnosis and management of congenital and gestational syphilis before and after the workshops.

**Table 2:** Distribution of the answers of the health professionals about diagnosis and management of congenital and gestational syphilis, according to successes and errors before and after the permanent education workshops, Londrina, PR, Brazil, 2014

Rated Knowledge	Stage of application of the questionnaire					p*				
	Before the Workshop (n = 102)					After the Workshop (n = 85)				
	Successes			Errors		Successes			Errors	
	n	%	n	%		n	%	n	%	
Difference between serologies FTA-Abs e VDRL (NR^†^ = 4)	77	75.5	21	20.6		85	100.0	-	-	< 0.001
Number of exams VDRL^‡^ mandatory in the PN according to the Rede Mãe Paranaense	79	77.5	23	22.5		85	100.0	-	-	< 0.001
Conduct when the result of the VDRL^‡^ is reagent	08	7.8	94	92.2		45	47.1	40	52.9	< 0.001
Conduct when the diagnosis of gestational syphilis is confirmed in the PN^§^	73	71.6	29	28.4		78	91.8	07	8.2	0.461
Procedures for controlling and monitoring gestational syphilis	55	53.9	47	46.1		77	90.6	08	9.4	0.002
Syphilis Notification	09	8.8	93	91.2		25	29.4	60	70.6	< 0.001
Therapeutic scheme recommended for primary syphilis (NR^†^ = 2)	58	56.9	42	42.0		84	98.8	01	1.2	< 0.001
Therapeutic scheme recommended for secondary syphilis (NR^†^ = 5)	56	57.7	41	42.2		76	89.4	09	10.6	< 0.001
Therapeutic scheme recommended for tertiary syphilis(NR^†^ = 5)	63	64.9	34	35.1		85	100.0	-	-	< 0.001
Monitoring of the RN of the mother diagnosed with syphilis during pregnancy	56	54.9	46	45.1		81	95.3	04	4.7	< 0.001

*McNemar test for correlated frequencies; ^†^Number of unanswered questions that modified the sample total; ^‡^VDRL - *Venereal Disease Research Laboratory* - Non-treponemal test; ^§^PN - prenatal

The number of people who answered the questionnaire after the intervention was lower (n = 85) to the number of participants who responded before (n = 102). The average successes were 53% before the intervention and reached 74,3% after (p < 0,001).

Regarding the notification, Table 3 distributes the occurrence of registered cases of gestational and congenital syphilis between 2007 and 2015. 

**Table 3:** Distribution of cases of gestational and congenital syphilis reported* between 2007 and 2015, Londrina, PR, Brazil, 2016

Year/cases	Gestational Syphilis	Congenital syphilis	Transmission %
2007	04	05	125.0^†^
2008	08	12	150.0^†^
2009	15	17	128.0^†^
2010	27	17	63.0
2011	29	20	69.0
2012	49	40	81.6
2013	68	51	75.0
2014	104	35	33.7
2015	122	49	40.2
Total	426	246	57.7

*Source: Sinan/Datasus/MS/Tabwin/Arquivo Sifgenet (2016); ^†^Underreporting

It is observed that between 2007 and 2009 there was underreporting of gestational syphilis. As of 2010, there is an increase in the notification of the problem in pregnant women, but vertical transmission remained high until 2013. In 2014 and 2015, after the educational intervention, there was a reduction in mother-to-child transmission rates in approximately 38%. 

With regards to mortality, Table 4 shows the number of fetal and infant deaths due to this condition.

**Table 4:** Number of fetal and infant deaths by congenital syphilis reported* between 2009 and 2015, Londrina, PR, Brazil, 2016

Year	Fetal Deaths	Infant Deaths	Total
2009	NI^†^	01	01
2010	NI^†^	01	01
2011	NI^†^	02	02
2012	02	02	04
2013	02	03^‡^	05
2014	05	-	05
2015	04	-	04
Total	13	09	22

*Source: SIM/Datasus/MS.CSIE/GVE/DVS/MAS (2016); ^†^Not investigated; ^‡^One death due to late congenital neurosyphilis (A504) and two deaths from unspecified congenital syphilis (A509).

Although the occurrence of child mortality from syphilis has been recorded since 2009, fetal deaths from this cause have only been investigated since 2012. The specific mortality rate for congenital syphilis reached its peak in 2013, with 41.7 cases every 100,000 live births. However, although the proportion of fetal deaths increased during the two years following the intervention (2014 and 2015), there was no record of child deaths due to this item in the same period of time.

## Discussion 

Overall, the results showed important changes and improvements, both in the professionals’ responses to diagnosis and management of gestational and congenital syphilis after educational intervention (75% of correct answers), and in the detection of syphilis in pregnant women - 68 cases in 2013 for 122 in 2015 - and reduction in vertical transmission (35% between 2013 and 2015). These results support the proposal made by the Pan American Health Organization-PAHO(5), encouraging the introduction of training programs in the workplace, with the aim of improving the quality of patient care and safety, and in this study it was extended to SUS users and the community. 

It should be noted that prior to the intervention, the health professionals surveyed did not present satisfactory knowledge about the measures recommended by the MS and Rede Mãe Paranaense in syphilis prevention and control, since the average number of correct answers was 53%. Insufficient knowledge of the correct measures to control and prevent the transmission of syphilis reflects the reality of other Brazilian municipalities^2^ ^,^ ^6^ ^-^ ^9^, showing that the health professionals presented insufficient technical qualification to face the problem of syphilis in prenatal care^10^ ^-^ ^15^. 

To ensure agility in the diagnosis of syphilis, in its confirmation, and in accounting for the number of VDRL tests performed in prenatal care - an important indicator that measures the quality of care^16^ ^-^ ^18^ − An internal flow of communication between the laboratory and the UBS was instituted in Londrina. Before the training, only 7.8% of the professionals knew this flow, although it had already been in practice for five months. Faced with this reality, it is understood that permanent education actions and changes in the work process must have a planned continuity^18^, because their punctual action informs and updates the professionals, but the change of conduct in practice needs monitoring and correction of errors as learning, not punishment, reinforcing self-analysis and self-management activities of the health teams^19^ ^-^ ^20^. 

Although prenatal care is recognized as a very important area for the prevention of vertical transmission of syphilis^21^, directly influencing the quality indexes that affect the health of the pregnant woman and the fetus and / or newborn^10^ ^,^ ^22^, about 30% of the professionals were unaware of the need to initiate immediate treatment of the pregnant women and to call in their sexual partners through positive VDRL before the intervention. Only for this question did the increase in correct responses after training not significantly improve participants’ knowledge. In Fortaleza-CE*, half of the interviewed professionals stated that they would request the VDRL for the partner and would only deal with the result^13^. A similar study carried out in Recife-PE* showed a value of 38% for the same index^20^, closer to the one found in Londrina. The data corroborate those pointed out in a similar survey carried in Rio de Janeiro-RJ*, in which approximately 40% of medical professionals and nurses reported having difficulty discussing syphilis with sexual partners^9^. 

The lack of recruitment and orientation of the partners and the difficulty of the health professionals in using the recommended therapeutic scheme for these cases - concomitant with the pregnant women - has been evidenced in several studies^9^ ^,^ ^15^ ^-^ ^16^, leading to the understanding that this protocol standard has not yet been fully assimilated, causing errors at the time of care and provoking inadequate treatments that reflect in the elevation of cases of congenital syphilis^20^.

Another recent problem is the national shortage of penicillin due to the lack of specific raw material for its production in the world market. In 2015, the MS published the Joint Information Note nº 109/2015/GAB/SVS/MS, which guides the prioritization of penicillin G benzathine for syphilis in pregnant women and crystalline penicillin for congenital syphilis in the country and alternatives for the treatment of syphilis. The greatest difficulty found is that the recommended second-choice antibiotics available in Brazilian SUS (Doxycycline and Ceftriaxone), in order to treat cases of acquired syphilis and partners of pregnant women, have a dosage during 8 to 15 days, further impairing adherence to the complete therapeutic scheme, which increases the chances of developing resistance regarding *Treponema pallidum* ^23^. 

It is emphasized that this situation results in treatment failures and consequent reinfections in cases in which the pregnant women are correctly treated, but their partners are not. Possibly the increase of the vertical transmission rate in 6,5% of 2014 to 2015 found in this study resulted from this fact, since the shortage in the network limited the use of the first choice drug to the affected binomial, as well as the fact that the interventions started in 2013, when the drug was not yet missing, reduced the vertical transmission rate in 41.3 % in the year 2014.

Regarding the notification, although the significant increase of adequate responses after the intervention, the percentage of correct answers was considered low since it did not reach 30%. This is added to the difficulty in identifying gestational and congenital syphilis as notifiable diseases^13^ and the incorporation of the notification of acquired syphilis to the work routines, fundamental for the disruption of the chain of transmission and control of the disease^21^. The search for the eradication of syphilis has been a great challenge for health professionals, health authorities and society at large over the years, and precisely for this reason this concept needs to be better addressed in health services^15^. 

In addition, misconceptions about the correct treatment of syphilis according to staging of the disease^6^ ^,^ ^9^ ^,^ ^13^, as well as in conducting VDRL titration for control of the treatment, as well as recruitment of sexual partners for testing, counseling and appropriate treatment, point out that the de-structuring of the work process favors the occurrence of many missed opportunities for diagnosis and intervention that would enable the prevention of vertical transmission^4^ ^,^ ^7^.

In addition, the index of erroneous responses in this study to questions about the syndromic approach was 40%, a scenario also found in Rio de Janeiro-RJ when 47% of professionals reported having some difficulty in the practice of adequate syphilis management^7^. Other studies conducted in Brazil^6^ ^,^ ^21^, in China^17^, in Switzerland^12^ and in the United States of America^11^ reinforced the same flaws and the reappearance of congenital syphilis today.

It is emphasized that vertical transmission causes serious consequences to these newborn infants, due to the failures in primary prevention^13^ ^-^ ^14^, which have sequelae (largely irreversible), as well as prematurity^7^ ^,^ ^10^ and put a burden on the health system^20^ ^-^ ^21^. The understanding of the healthcare network organization with reference institutions directed to the pediatric infectious diseases outpatient clinic and the counter-referral from the maternity to the UBS by e-mail, probably influenced the absence of syphilis infant mortality in the two years following the intervention (2014 and 2015), as a result of improved pre- and postnatal care. 

In this study, evidence of underreporting is highlighted, when between 2007 and 2009 cases of congenital syphilis exceeded the cases detected in pregnant women. These data reflect the reality of the various states of Brazil^21^ and from other countries such as Mongolia and South Africa^24^. However, there was an improvement in the access to the diagnosis of pregnant women, since the rate of detection of gestational syphilis after educational interventions increased from 9.4 in 2013 to 16.7 cases per thousand live births in 2015. In this way, the adhesion of professionals and the commitment to replicate the workshops in loco in 92.3% of UBSs in Londrina, indicates that the process of permanent education in AB, in partnership with the health team and municipal management, strengthened the practice in the prevention and control of syphilis.

Due to the fact that it is a quasi-experimental study, it can be considered a methodological limitation the absence of a control and follow-up group. These options were consciously made, considering that all health professionals who attended syphilis diagnosis and management workshops adhered to the project and were closely monitored by regional health managers who provided support throughout the ongoing education process, which still continues. Other researches used the same method and concluded that skill development after training is statistically significant when compared to the control group and that skills are largely maintained six months after training^14^ ^,^ ^25^.

## Conclusion

The educational intervention interfered in improving the early detection of gestational syphilis and led to a reduction in the vertical transmission rate, as well as may have contributed to eliminating syphilis-specific mortality in children under one year in 2014 and 2015.

## References

[1] WHO (2012). Investment case for eliminating mother-to-child transmission of syphilis:
promoting better maternal and child health and stronger health systems
[Internet]..

[2] Ministério da Saúde (BR). Secretaria de Vigilância em Saúde (2014). Programa Nacional de DST e Aids. Transmissão Vertical do HIV e Sífilis:
Estratégias para Redução e Eliminação.

[3] Fertonani HP, Pires DEP, Biff D, Scherer MDA (2015). The health care model: concepts and challenges for primary health care
in Brazil. Ciênc Saúde Col..

[4] Domingues RMS, Saraceni V, Hartz ZMA, Leal MC (2013). Congenital syphilis: a sentinel event in antenatal care
quality. Rev Saúde Pública.

[5] Cometto MC, Gómez PF, Sasso GTMD, Grajales RAZ, Cassiani SHDB, Morales CF (2011). Enfermería y seguridad de los pacientes. Washington, DC: Organización
Panamericana de la Salud.

[6] Gomes SF (2013). Conhecimentos, atitudes e práticas dos médicos e enfermeiros das unidades de
saúde da família sobre sífilis em gestantes na cidade do Recife- PE.

[7] Rodrigues CS, Guimarães MDC, César CC (2008). Missed opportunities for congenital syphilis and HIV perinatal
transmission prevention. Rev Saúde Pública.

[8] Ministério da Saúde (BR). Secretaria de Vigilância em Saúde. Departamento
de DST, Aids e Hepatites Virais (2015). Boletim Epidemiológico - Sífilis Ano IV.

[9] Domingues RMSM, Lauria LM, Saraceni V, Leal MC (2013). Treatment of syphilis during pregnancy: knowledge, practices and
attitudes of health care professionals involved in antenatal care of the Unified
Health System (SUS) in Rio de Janeiro City. Ciênc Saúde Col.

[10] Blencowe H, Cousens S, Kamb M, Berman S, Lawn JE (2011). Lives saved tool supplement detection and treatment of syphilis in
pregnancy to reduce syphilis related stillbirths and neonatam
mortality. BMC Public Health.

[11] Bowen V, Su J, Torrone E, Kidd S, Weinstock H (2015). Increase in Incidence of Congenital Syphilis − United States,
2012-2014. Morb Mortal Wkly Rep.

[12] Meyer Sauteur PM, Trück J, Bosshard PP, Tomaske M, Morán Cadenas F, Lautenschlager S, Goetschel P (2012). Congenital syphilis in Switzerland: gone, forgotten, on the
return. Swiss Med Wkly.

[13] Andrade RFV, Lima NBG, Araújo MAL, Silva DMA, Melo SP (2011). Nurses’s Knowledge about the Management of Pregnant with Positive
VDRL. DST − J Bras Doenças Sex Transm.

[14] Silva DMA, Araújo MAL, Silva RM, Andrade RFV, Moura HJ, Esteves ABB (2014). Knowledge of healthcare professionals regarding the vertical
transmission of syphilis in Fortaleza-CE, Brazil. Texto Contexto - Enferm.

[15] Owiredu MN, Newman L, Nzomo T, Kafando GC, Sanni S, Shaffer N (2015). Elimination of mother-to-child transmission of HIV and syphilis: a
dual approach in the African Region to improve quality of antenatal care and
integrated disease control. Int J Gynecol Obstet.

[16] Todd CS, Ahmadzai M, Smith JM, Siddiqui H, Ghazanfar SA, Strathdee SA (2008). Attitudes and practices of obstetric care providers in Kabul,
Afghanistan regarding antenatal testing for sexually transmitted
infection. J Obstet Gynecol Neonatal Nurs.

[17] Wu D, Hawkes S, Buse K (2015). Prevention of mother-to-child transmission of syphilis and HIV in
China: what drives political prioritization and what can this tell us about
promoting dual elimination?. Int J Gynecol Obstet.

[18] Hussey J, Mitchell L, Hew Y, Foster K, Waldram A (2014). Preventing congenital syphilis: a regional audit of syphilis in
pregnant women seen in Genitourinary Medicine services. Int J STD AIDS.

[19] Ceccim RB, Armani TB (2001). Educação na saúde coletiva. Divulg Saúde Debate.

[20] Larson BA, Lembela-Bwalya D, Bonawitz R, Hammond EE, Thea DM, Herlihy J (2014). Finding a needle in the haystack: the costs and cost-effectiveness of
syphilis diagnosis and treatment during pregnancy to prevent congenital syphilis
in Kalomo District of Zambia. Plos One.

[21] Domingues RMSM, Szwarcwald CL, Souza PRB, Leal MC (2014). Prevalence of syphilis in pregnancy and prenatal syphilis testing in
Brazil: birth in Brazil study. Rev Saúde Pública.

[22] WHO (2012). Investment case for eliminating mother-to-child transmission of syphilis:
promoting better maternal and child health and stronger health systems.

[23] Stamm LV (2015). Syphilis: antibiotic treatment and resistance. Epidemiol Infect.

[24] Shahrook S, Mori R, Ochirbat T, Gomi H (2014). Strategies of testing for syphilis during pregnancy. Cochrane Database Syst Rev.

[25] Carmona-Navarro MC, Pichardo-Martínez MC (2012). Attitudes of nursing professionals towards suicidal behavior:
influence of emotional intelligence. Rev. Latino-Am. Enfermagem.

